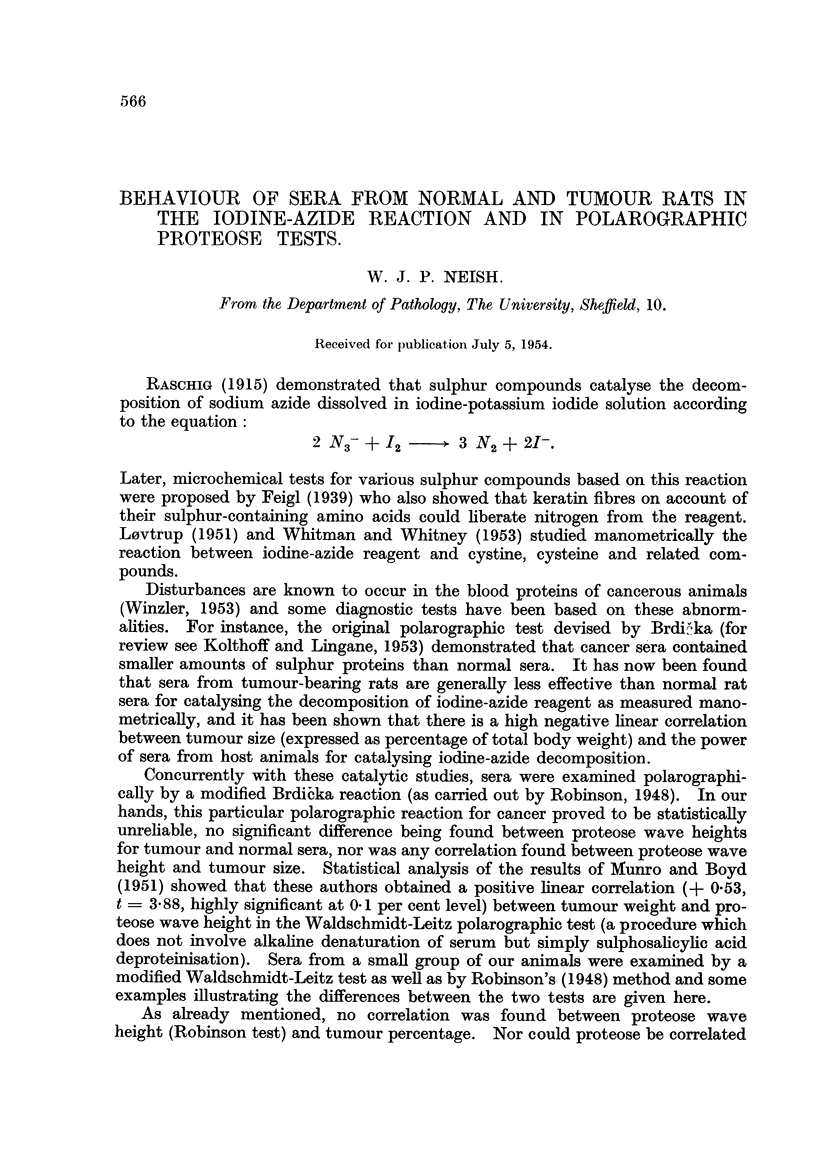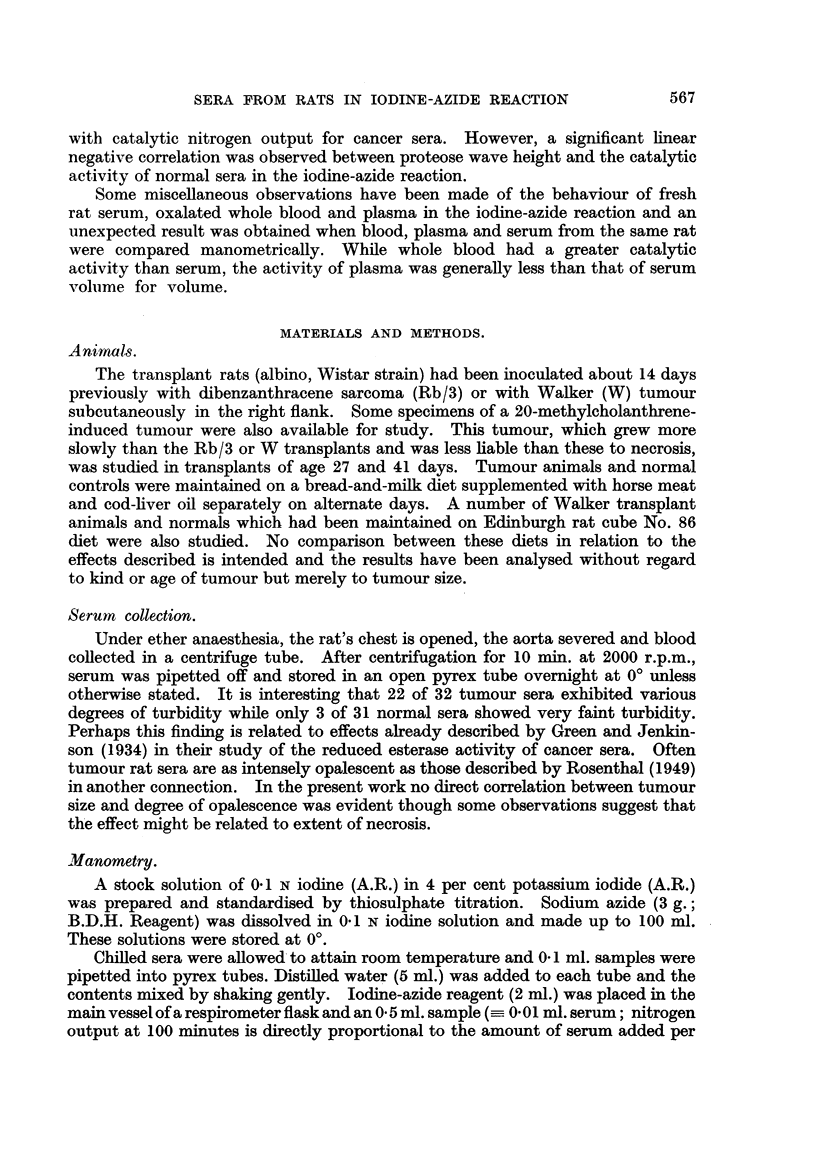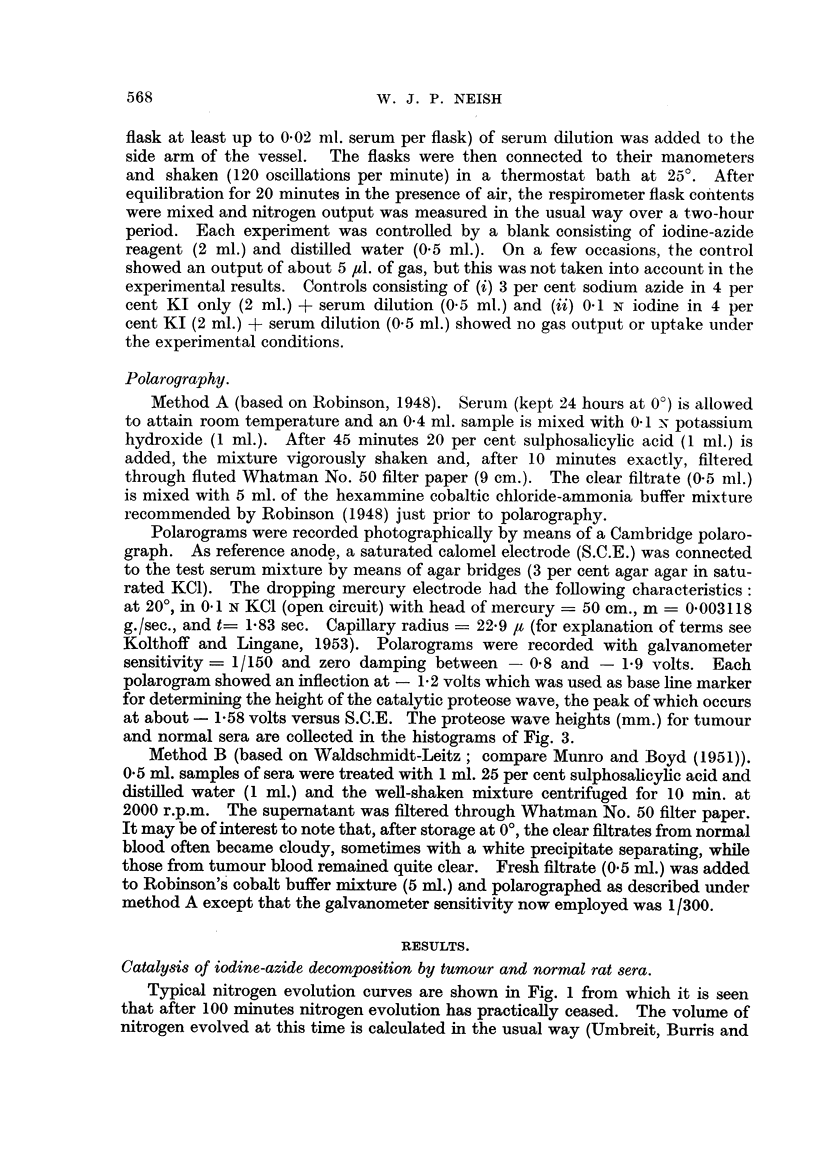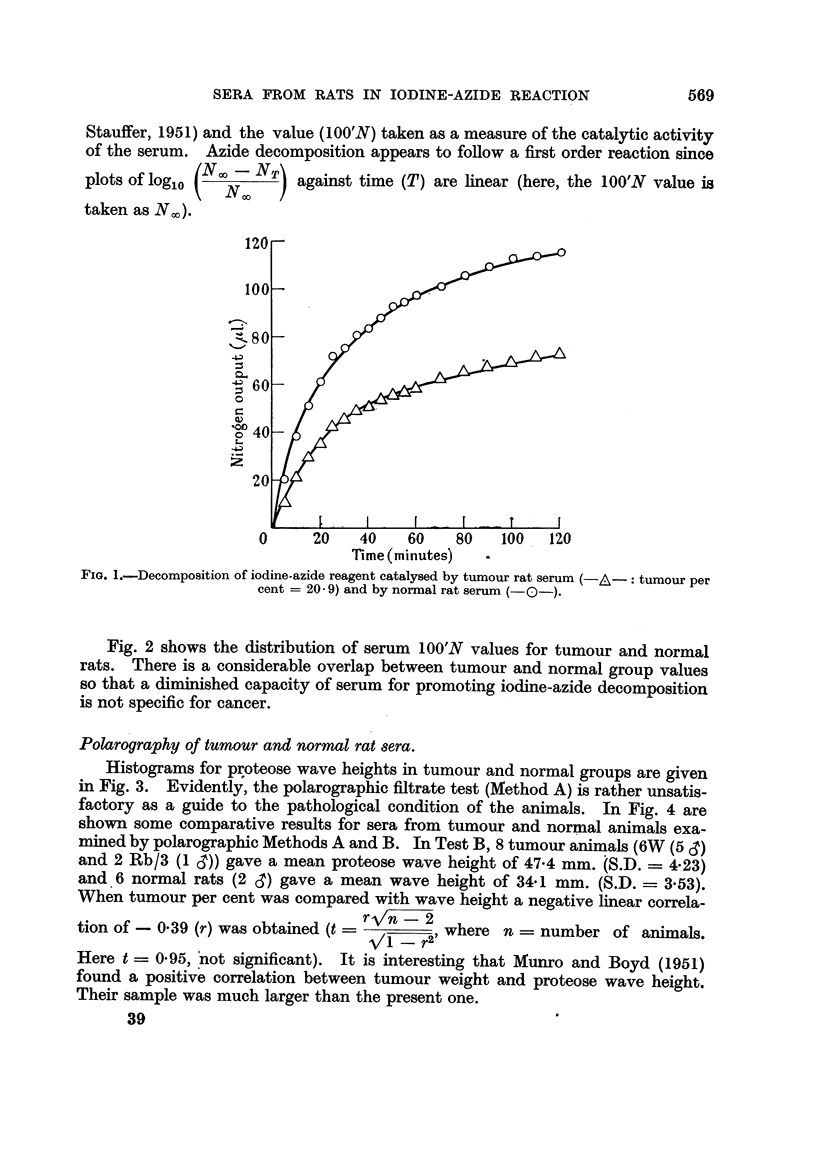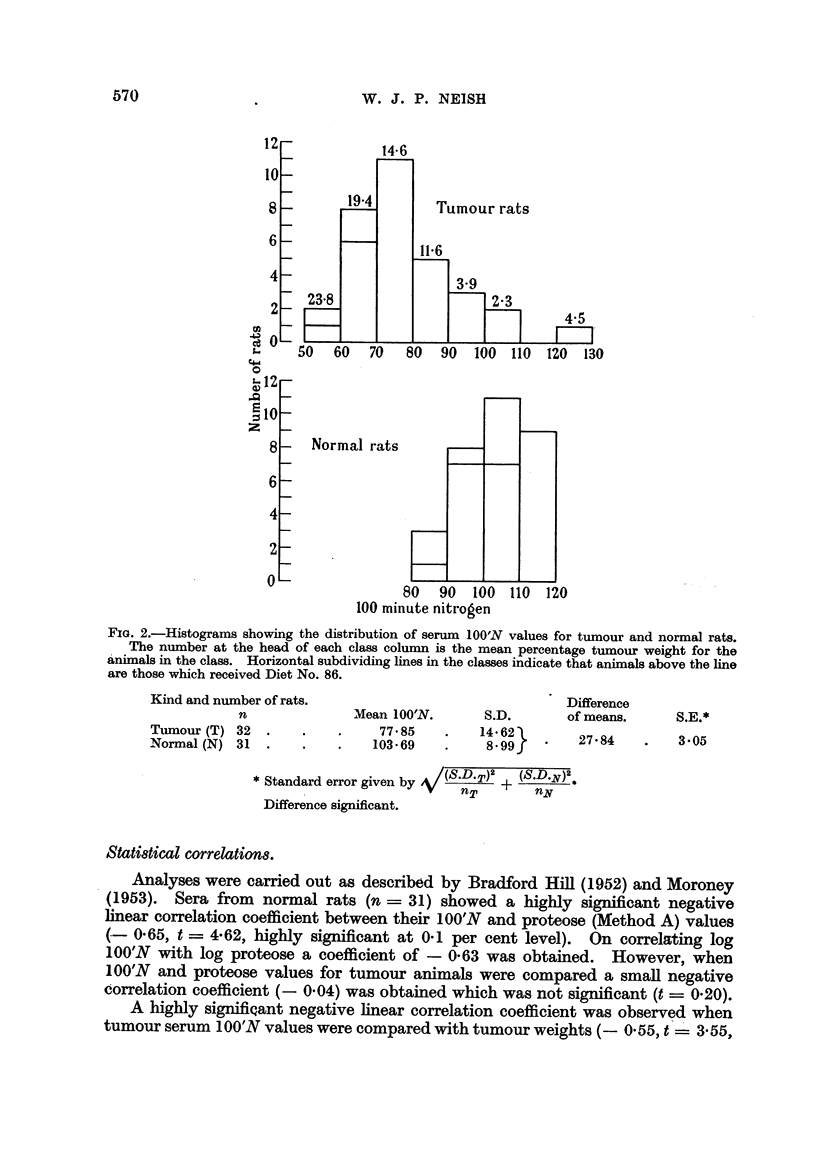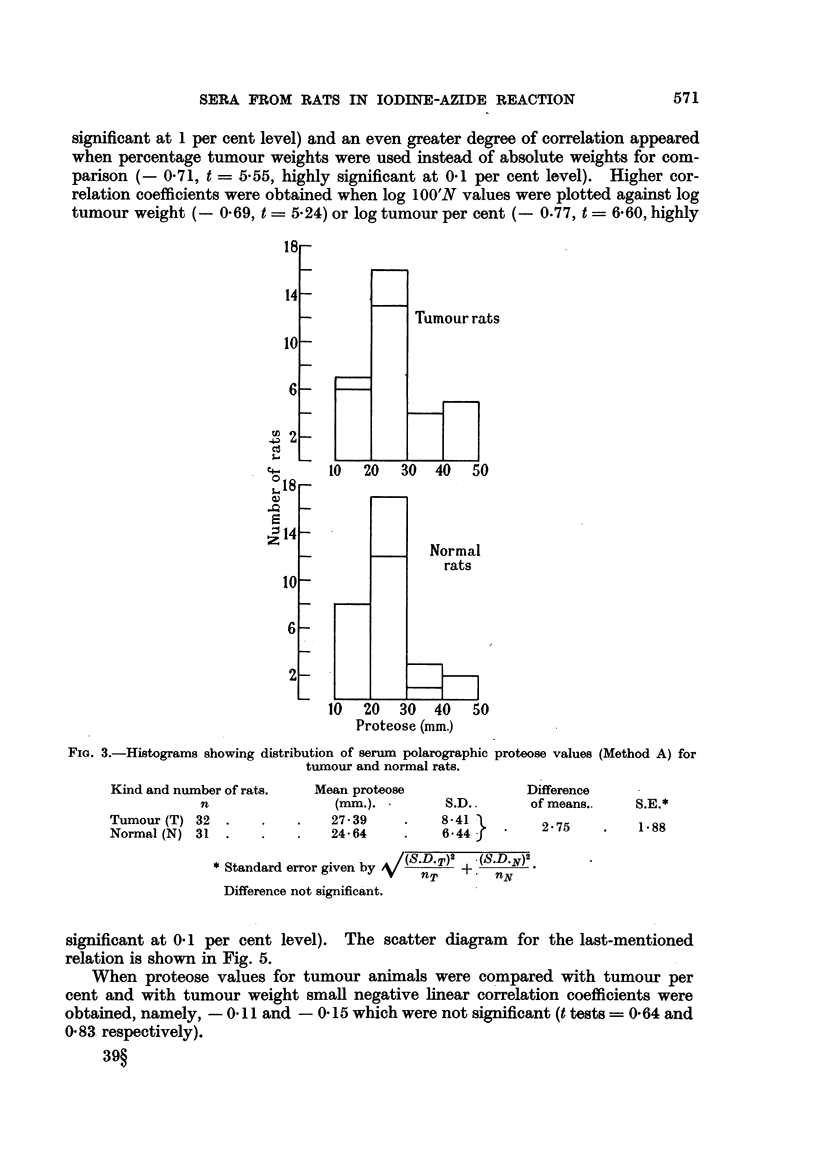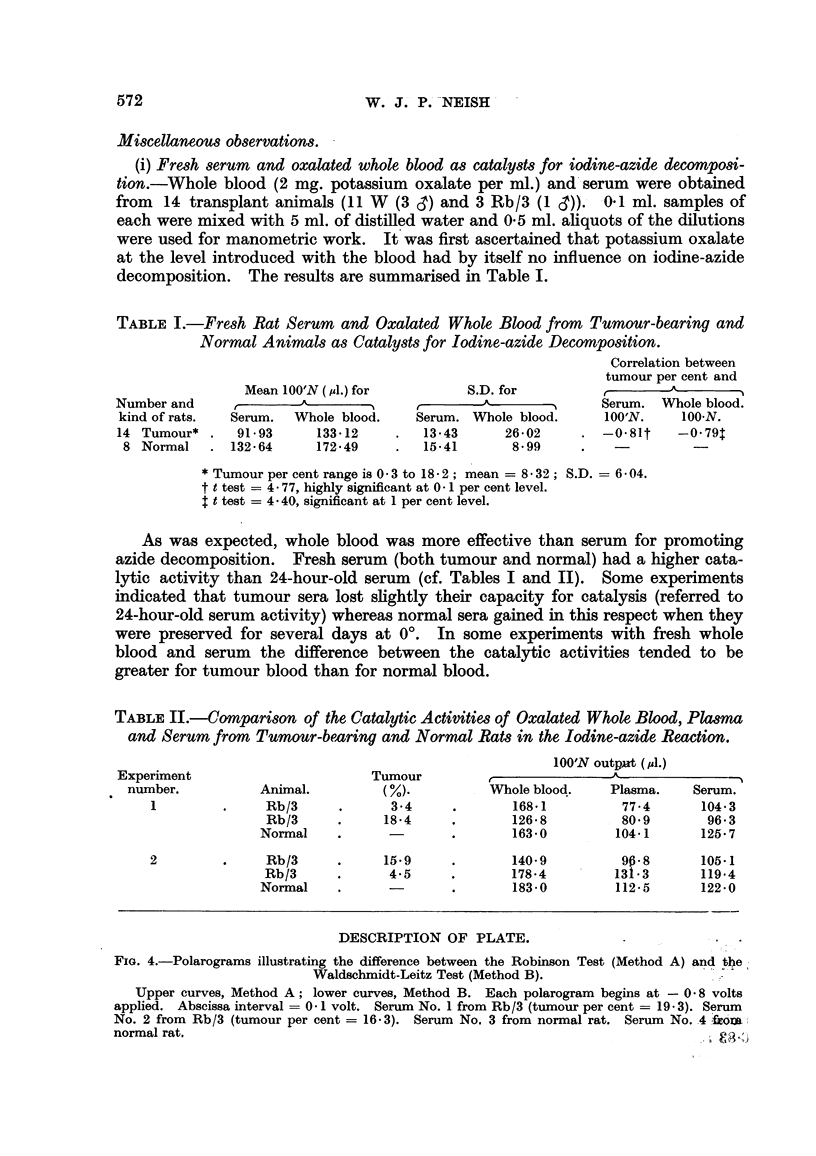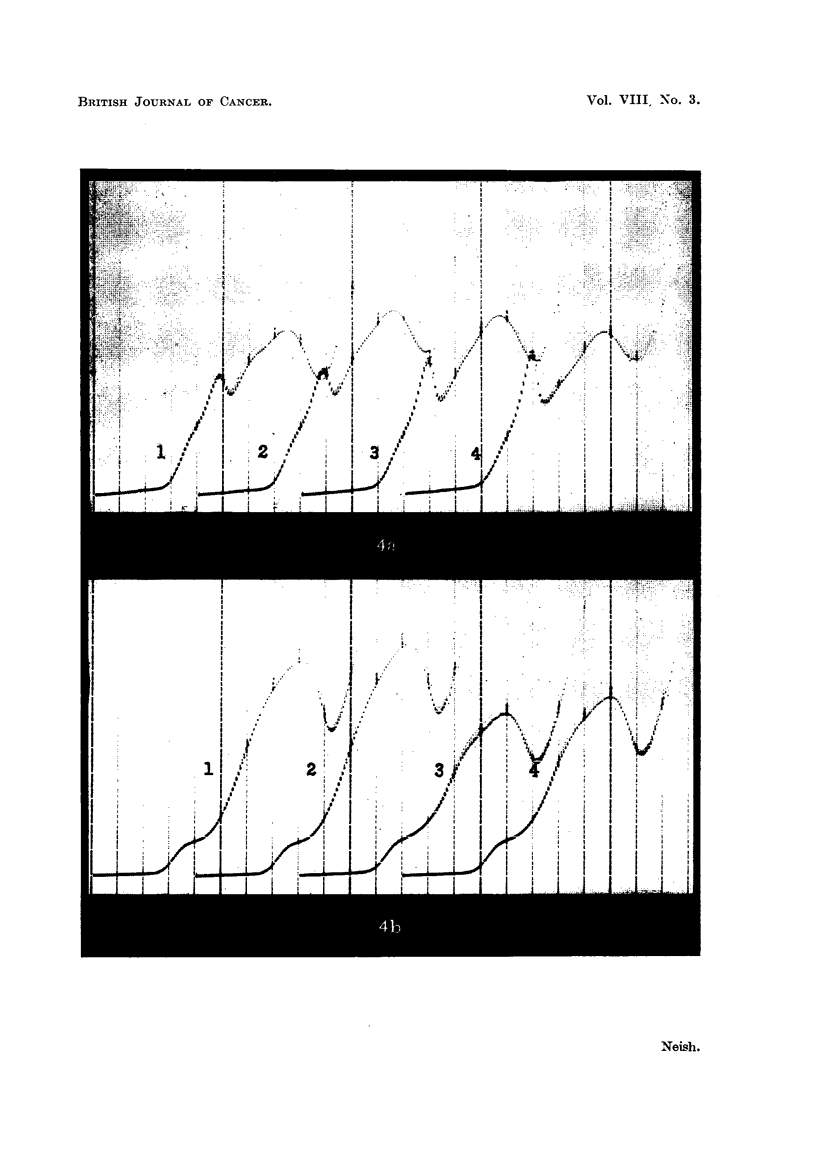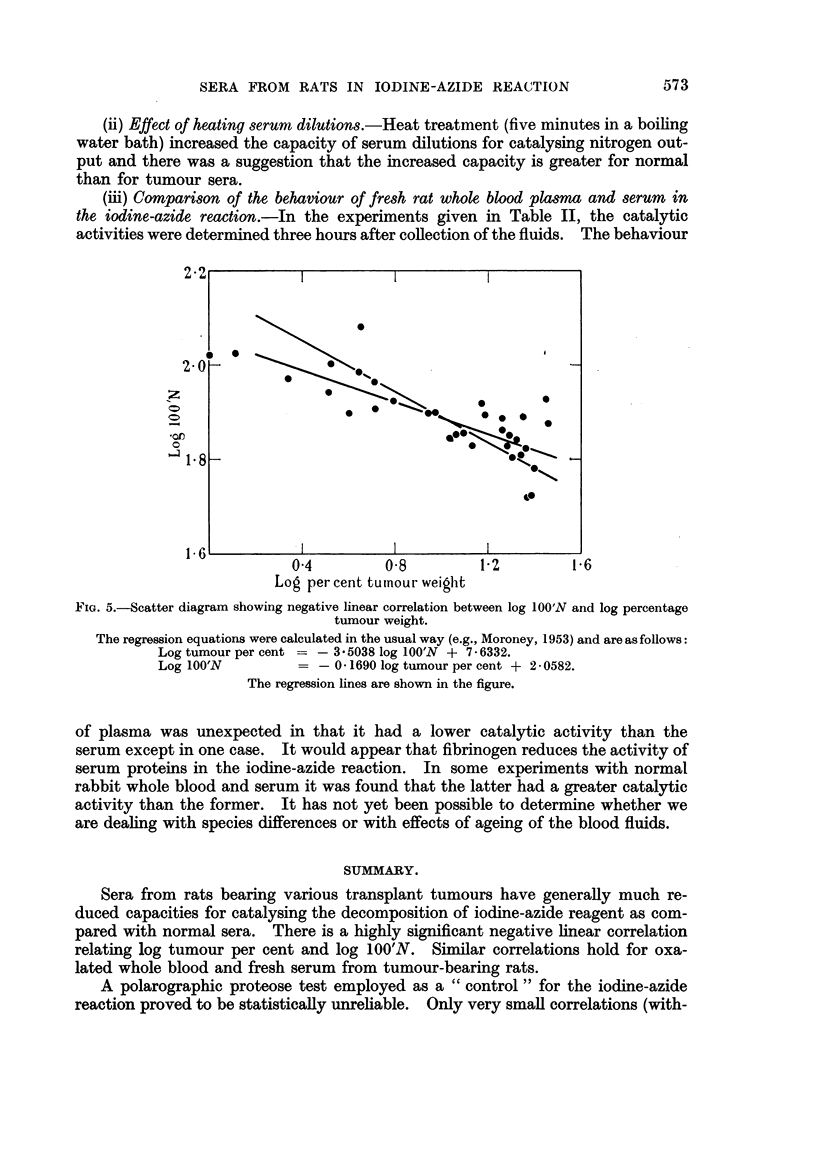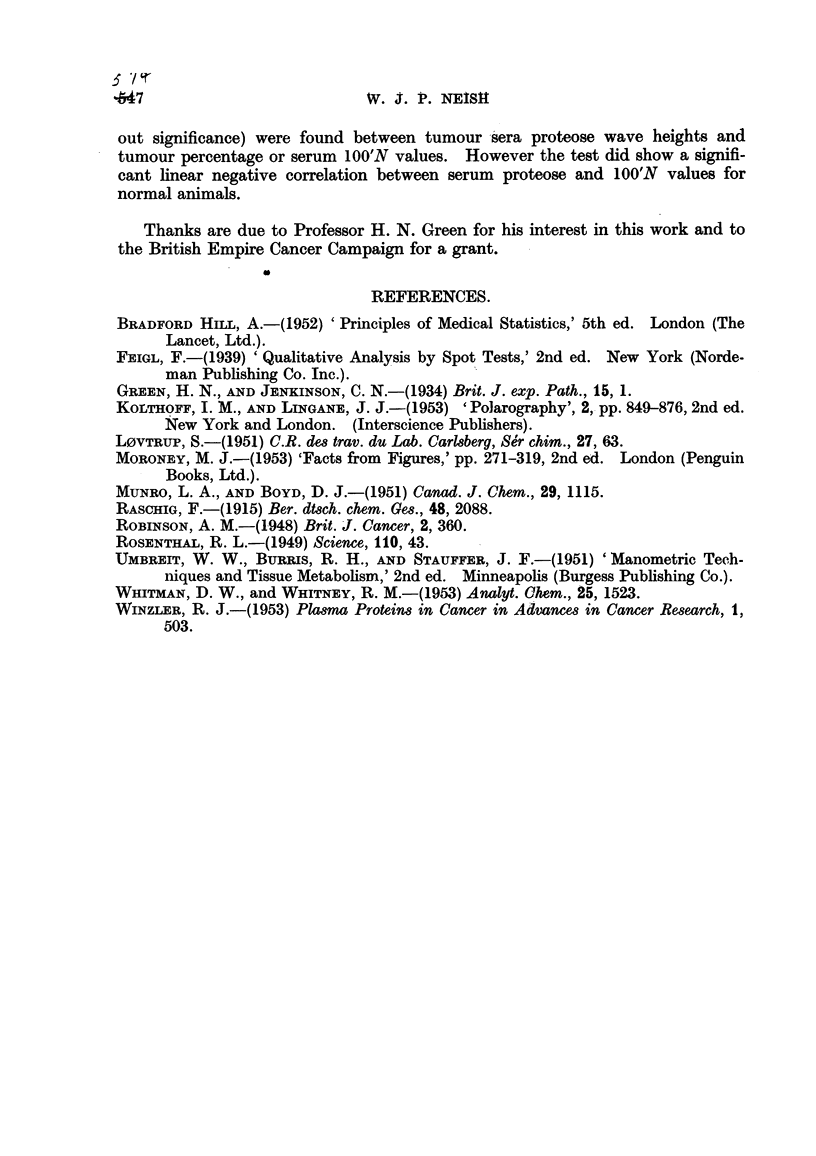# Behaviour of Sera from Normal and Tumour Rats in the Iodine-Azide Reaction and in Polarographic Proteose Tests

**DOI:** 10.1038/bjc.1954.61

**Published:** 1954-09

**Authors:** W. J. P. Neish

## Abstract

**Images:**


### BEHAVIOUR OF SERA FROM NORMAL AND TUMOUR RATS IN THE IODINE-AZIDE REACTION AND IN POLAROGRAPHIC PROTEOSE TESTS.

W. J. P. NEISH.

*From the Department of Pathology, The University, Sheffield,* 10.

Received for publication July 5, 1954.

RASCHIG (1915) demonstrated that sulphur compounds catalyse the decomposition of sodium azide dissolved in iodine-potassium iodide solution according to the equation :

$$2\ N_3^- + I_2 \longrightarrow 3\ N_2 + 2I^-.$$

Later, microchemical tests for various sulphur compounds based on this reaction were proposed by Feigl (1939) who also showed that keratin fibres on account of their sulphur-containing amino acids could liberate nitrogen from the reagent. Løvtrup (1951) and Whitman and Whitney (1953) studied manometrically the reaction between iodine-azide reagent and cystine, cysteine and related compounds.

Disturbances are known to occur in the blood proteins of cancerous animals (Winzler, 1953) and some diagnostic tests have been based on these abnormalities. For instance, the original polarographic test devised by Brdička (for review see Kolthoff and Lingane, 1953) demonstrated that cancer sera contained smaller amounts of sulphur proteins than normal sera. It has now been found that sera from tumour-bearing rats are generally less effective than normal rat sera for catalysing the decomposition of iodine-azide reagent as measured manometrically, and it has been shown that there is a high negative linear correlation between tumour size (expressed as percentage of total body weight) and the power of sera from host animals for catalysing iodine-azide decomposition.

Concurrently with these catalytic studies, sera were examined polarographically by a modified Brdička reaction (as carried out by Robinson, 1948). In our hands, this particular polarographic reaction for cancer proved to be statistically unreliable, no significant difference being found between proteose wave heights for tumour and normal sera, nor was any correlation found between proteose wave height and tumour size. Statistical analysis of the results of Munro and Boyd (1951) showed that these authors obtained a positive linear correlation (+ 0·53, $t = 3{\cdot}88$, highly significant at 0·1 per cent level) between tumour weight and proteose wave height in the Waldschmidt-Leitz polarographic test (a procedure which does not involve alkaline denaturation of serum but simply sulphosalicylic acid deproteinisation). Sera from a small group of our animals were examined by a modified Waldschmidt-Leitz test as well as by Robinson's (1948) method and some examples illustrating the differences between the two tests are given here.

As already mentioned, no correlation was found between proteose wave height (Robinson test) and tumour percentage. Nor could proteose be correlated with catalytic nitrogen output for cancer sera. However, a significant linear negative correlation was observed between proteose wave height and the catalytic activity of normal sera in the iodine-azide reaction.

Some miscellaneous observations have been made of the behaviour of fresh rat serum, oxalated whole blood and plasma in the iodine-azide reaction and an unexpected result was obtained when blood, plasma and serum from the same rat were compared manometrically. While whole blood had a greater catalytic activity than serum, the activity of plasma was generally less than that of serum volume for volume.

### MATERIALS AND METHODS.

*Animals.*

The transplant rats (albino, Wistar strain) had been inoculated about 14 days previously with dibenzanthracene sarcoma (Rb/3) or with Walker (W) tumour subcutaneously in the right flank. Some specimens of a 20-methylcholanthrene-induced tumour were also available for study. This tumour, which grew more slowly than the Rb/3 or W transplants and was less liable than these to necrosis, was studied in transplants of age 27 and 41 days. Tumour animals and normal controls were maintained on a bread-and-milk diet supplemented with horse meat and cod-liver oil separately on alternate days. A number of Walker transplant animals and normals which had been maintained on Edinburgh rat cube No. 86 diet were also studied. No comparison between these diets in relation to the effects described is intended and the results have been analysed without regard to kind or age of tumour but merely to tumour size.

*Serum collection.*

Under ether anaesthesia, the rat's chest is opened, the aorta severed and blood collected in a centrifuge tube. After centrifugation for 10 min. at 2000 r.p.m., serum was pipetted off and stored in an open pyrex tube overnight at 0° unless otherwise stated. It is interesting that 22 of 32 tumour sera exhibited various degrees of turbidity while only 3 of 31 normal sera showed very faint turbidity. Perhaps this finding is related to effects already described by Green and Jenkinson (1934) in their study of the reduced esterase activity of cancer sera. Often tumour rat sera are as intensely opalescent as those described by Rosenthal (1949) in another connection. In the present work no direct correlation between tumour size and degree of opalescence was evident though some observations suggest that the effect might be related to extent of necrosis.

*Manometry.*

A stock solution of 0·1 N iodine (A.R.) in 4 per cent potassium iodide (A.R.) was prepared and standardised by thiosulphate titration. Sodium azide (3 g.; B.D.H. Reagent) was dissolved in 0·1 N iodine solution and made up to 100 ml. These solutions were stored at 0°.

Chilled sera were allowed to attain room temperature and 0·1 ml. samples were pipetted into pyrex tubes. Distilled water (5 ml.) was added to each tube and the contents mixed by shaking gently. Iodine-azide reagent (2 ml.) was placed in the main vessel of a respirometer flask and an 0·5 ml. sample (≡ 0·01 ml. serum; nitrogen output at 100 minutes is directly proportional to the amount of serum added per flask at least up to 0·02 ml. serum per flask) of serum dilution was added to the side arm of the vessel. The flasks were then connected to their manometers and shaken (120 oscillations per minute) in a thermostat bath at 25°. After equilibration for 20 minutes in the presence of air, the respirometer flask contents were mixed and nitrogen output was measured in the usual way over a two-hour period. Each experiment was controlled by a blank consisting of iodine-azide reagent (2 ml.) and distilled water (0·5 ml.). On a few occasions, the control showed an output of about 5 $\mu$l. of gas, but this was not taken into account in the experimental results. Controls consisting of (*i*) 3 per cent sodium azide in 4 per cent KI only (2 ml.) + serum dilution (0·5 ml.) and (*ii*) 0·1 N iodine in 4 per cent KI (2 ml.) + serum dilution (0·5 ml.) showed no gas output or uptake under the experimental conditions.

*Polarography.*

Method A (based on Robinson, 1948). Serum (kept 24 hours at 0°) is allowed to attain room temperature and an 0·4 ml. sample is mixed with 0·1 N potassium hydroxide (1 ml.). After 45 minutes 20 per cent sulphosalicylic acid (1 ml.) is added, the mixture vigorously shaken and, after 10 minutes exactly, filtered through fluted Whatman No. 50 filter paper (9 cm.). The clear filtrate (0·5 ml.) is mixed with 5 ml. of the hexammine cobaltic chloride-ammonia buffer mixture recommended by Robinson (1948) just prior to polarography.

Polarograms were recorded photographically by means of a Cambridge polarograph. As reference anode, a saturated calomel electrode (S.C.E.) was connected to the test serum mixture by means of agar bridges (3 per cent agar agar in saturated KCl). The dropping mercury electrode had the following characteristics: at 20°, in 0·1 N KCl (open circuit) with head of mercury = 50 cm., m = 0·003118 g./sec., and $t$= 1·83 sec. Capillary radius = 22·9 $\mu$ (for explanation of terms see Kolthoff and Lingane, 1953). Polarograms were recorded with galvanometer sensitivity = 1/150 and zero damping between − 0·8 and − 1·9 volts. Each polarogram showed an inflection at − 1·2 volts which was used as base line marker for determining the height of the catalytic proteose wave, the peak of which occurs at about − 1·58 volts versus S.C.E. The proteose wave heights (mm.) for tumour and normal sera are collected in the histograms of Fig. 3.

Method B (based on Waldschmidt-Leitz ; compare Munro and Boyd (1951)). 0·5 ml. samples of sera were treated with 1 ml. 25 per cent sulphosalicylic acid and distilled water (1 ml.) and the well-shaken mixture centrifuged for 10 min. at 2000 r.p.m. The supernatant was filtered through Whatman No. 50 filter paper. It may be of interest to note that, after storage at 0°, the clear filtrates from normal blood often became cloudy, sometimes with a white precipitate separating, while those from tumour blood remained quite clear. Fresh filtrate (0·5 ml.) was added to Robinson's cobalt buffer mixture (5 ml.) and polarographed as described under method A except that the galvanometer sensitivity now employed was 1/300.

### RESULTS.

*Catalysis of iodine-azide decomposition by tumour and normal rat sera.*

Typical nitrogen evolution curves are shown in Fig. 1 from which it is seen that after 100 minutes nitrogen evolution has practically ceased. The volume of nitrogen evolved at this time is calculated in the usual way (Umbreit, Burris and

Stauffer, 1951) and the value (100′$N$) taken as a measure of the catalytic activity of the serum. Azide decomposition appears to follow a first order reaction since plots of $\log_{10}\left(\frac{N_\infty - N_T}{N_\infty}\right)$ against time ($T$) are linear (here, the 100′$N$ value is taken as $N_\infty$).

FIG. 1.—Decomposition of iodine-azide reagent catalysed by tumour rat serum (—△— : tumour per cent = 20·9) and by normal rat serum (—○—).

Fig. 2 shows the distribution of serum 100′$N$ values for tumour and normal rats. There is a considerable overlap between tumour and normal group values so that a diminished capacity of serum for promoting iodine-azide decomposition is not specific for cancer.

*Polarography of tumour and normal rat sera.*

Histograms for proteose wave heights in tumour and normal groups are given in Fig. 3. Evidently, the polarographic filtrate test (Method A) is rather unsatisfactory as a guide to the pathological condition of the animals. In Fig. 4 are shown some comparative results for sera from tumour and normal animals examined by polarographic Methods A and B. In Test B, 8 tumour animals (6W (5 ♂) and 2 Rb/3 (1 ♂)) gave a mean proteose wave height of 47·4 mm. (S.D. = 4·23) and 6 normal rats (2 ♂) gave a mean wave height of 34·1 mm. (S.D. = 3·53). When tumour per cent was compared with wave height a negative linear correlation of — 0·39 ($r$) was obtained ($t = \frac{r\sqrt{n-2}}{\sqrt{1-r^2}}$, where $n$ = number of animals. Here $t$ = 0·95, not significant). It is interesting that Munro and Boyd (1951) found a positive correlation between tumour weight and proteose wave height. Their sample was much larger than the present one.

FIG. 2.—Histograms showing the distribution of serum 100′*N* values for tumour and normal rats. The number at the head of each class column is the mean percentage tumour weight for the animals in the class. Horizontal subdividing lines in the classes indicate that animals above the line are those which received Diet No. 86.

| Kind and number of rats. | *n* | Mean 100′*N*. | S.D. | Difference of means. | S.E.* |
|---|---|---|---|---|---|
| Tumour (T) | 32 | 77·85 | 14·62 | 27·84 | 3·05 |
| Normal (N) | 31 | 103·69 | 8·99 | | |

* Standard error given by $\sqrt{\frac{(S.D._T)^2}{n_T} + \frac{(S.D._N)^2}{n_N}}$.

Difference significant.

*Statistical correlations.*

Analyses were carried out as described by Bradford Hill (1952) and Moroney (1953). Sera from normal rats ($n = 31$) showed a highly significant negative linear correlation coefficient between their 100′*N* and proteose (Method A) values (— 0·65, $t = 4{\cdot}62$, highly significant at 0·1 per cent level). On correlating log 100′*N* with log proteose a coefficient of — 0·63 was obtained. However, when 100′*N* and proteose values for tumour animals were compared a small negative correlation coefficient (— 0·04) was obtained which was not significant ($t = 0{\cdot}20$).

A highly significant negative linear correlation coefficient was observed when tumour serum 100′*N* values were compared with tumour weights (— 0·55, $t = 3{\cdot}55$, significant at 1 per cent level) and an even greater degree of correlation appeared when percentage tumour weights were used instead of absolute weights for comparison (— 0·71, $t = 5{\cdot}55$, highly significant at 0·1 per cent level). Higher correlation coefficients were obtained when log 100'$N$ values were plotted against log tumour weight (— 0·69, $t = 5{\cdot}24$) or log tumour per cent (— 0·77, $t = 6{\cdot}60$, highly

FIG. 3.—Histograms showing distribution of serum polarographic proteose values (Method A) for tumour and normal rats.

| Kind and number of rats. *n* | Mean proteose (mm.). | S.D. | Difference of means. | S.E.* |
|---|---|---|---|---|
| Tumour (T) 32 | 27·39 | 8·41 | 2·75 | 1·88 |
| Normal (N) 31 | 24·64 | 6·44 | | |

* Standard error given by $\sqrt{\frac{(S.D._T)^2}{n_T} + \frac{(S.D._N)^2}{n_N}}$.

Difference not significant.

significant at 0·1 per cent level). The scatter diagram for the last-mentioned relation is shown in Fig. 5.

When proteose values for tumour animals were compared with tumour per cent and with tumour weight small negative linear correlation coefficients were obtained, namely, — 0·11 and — 0·15 which were not significant ($t$ tests = 0·64 and 0·83 respectively).

*Miscellaneous observations.*

(i) ***Fresh serum and oxalated whole blood as catalysts for iodine-azide decomposition.***—Whole blood (2 mg. potassium oxalate per ml.) and serum were obtained from 14 transplant animals (11 W (3 ♂) and 3 Rb/3 (1 ♂)). 0·1 ml. samples of each were mixed with 5 ml. of distilled water and 0·5 ml. aliquots of the dilutions were used for manometric work. It was first ascertained that potassium oxalate at the level introduced with the blood had by itself no influence on iodine-azide decomposition. The results are summarised in Table I.

TABLE I.—*Fresh Rat Serum and Oxalated Whole Blood from Tumour-bearing and Normal Animals as Catalysts for Iodine-azide Decomposition.*

| Number and kind of rats. | Mean 100'N (μl.) for: Serum. | Mean 100'N (μl.) for: Whole blood. | S.D. for: Serum. | S.D. for: Whole blood. | Correlation between tumour per cent and: Serum. 100'N. | Correlation between tumour per cent and: Whole blood. 100·N. |
|---|---|---|---|---|---|---|
| 14 Tumour* | 91·93 | 133·12 | 13·43 | 26·02 | −0·81† | −0·79‡ |
| 8 Normal | 132·64 | 172·49 | 15·41 | 8·99 | — | — |

* Tumour per cent range is 0·3 to 18·2 ; mean = 8·32 ; S.D. = 6·04.
† *t* test = 4·77, highly significant at 0·1 per cent level.
‡ *t* test = 4·40, significant at 1 per cent level.

As was expected, whole blood was more effective than serum for promoting azide decomposition. Fresh serum (both tumour and normal) had a higher catalytic activity than 24-hour-old serum (cf. Tables I and II). Some experiments indicated that tumour sera lost slightly their capacity for catalysis (referred to 24-hour-old serum activity) whereas normal sera gained in this respect when they were preserved for several days at 0°. In some experiments with fresh whole blood and serum the difference between the catalytic activities tended to be greater for tumour blood than for normal blood.

TABLE II.—*Comparison of the Catalytic Activities of Oxalated Whole Blood, Plasma and Serum from Tumour-bearing and Normal Rats in the Iodine-azide Reaction.*

| Experiment number. | Animal. | Tumour (%). | 100'N output (μl.): Whole blood. | 100'N output (μl.): Plasma. | 100'N output (μl.): Serum. |
|---|---|---|---|---|---|
| 1 | Rb/3 | 3·4 | 168·1 | 77·4 | 104·3 |
| | Rb/3 | 18·4 | 126·8 | 80·9 | 96·3 |
| | Normal | — | 163·0 | 104·1 | 125·7 |
| 2 | Rb/3 | 15·9 | 140·9 | 96·8 | 105·1 |
| | Rb/3 | 4·5 | 178·4 | 131·3 | 119·4 |
| | Normal | — | 183·0 | 112·5 | 122·0 |

DESCRIPTION OF PLATE.

FIG. 4.—Polarograms illustrating the difference between the Robinson Test (Method A) and the Waldschmidt-Leitz Test (Method B).

Upper curves, Method A ; lower curves, Method B. Each polarogram begins at − 0·8 volts applied. Abscissa interval = 0·1 volt. Serum No. 1 from Rb/3 (tumour per cent = 19·3). Serum No. 2 from Rb/3 (tumour per cent = 16·3). Serum No. 3 from normal rat. Serum No. 4 from normal rat.

4a

4b

Neish.

(ii) *Effect of heating serum dilutions.*—Heat treatment (five minutes in a boiling water bath) increased the capacity of serum dilutions for catalysing nitrogen output and there was a suggestion that the increased capacity is greater for normal than for tumour sera.

(iii) *Comparison of the behaviour of fresh rat whole blood plasma and serum in the iodine-azide reaction.*—In the experiments given in Table II, the catalytic activities were determined three hours after collection of the fluids. The behaviour

FIG. 5.—Scatter diagram showing negative linear correlation between log 100'*N* and log percentage tumour weight.

The regression equations were calculated in the usual way (e.g., Moroney, 1953) and are as follows:
Log tumour per cent = $-$ 3·5038 log 100'*N* + 7·6332.
Log 100'*N* = $-$ 0·1690 log tumour per cent + 2·0582.

The regression lines are shown in the figure.

of plasma was unexpected in that it had a lower catalytic activity than the serum except in one case. It would appear that fibrinogen reduces the activity of serum proteins in the iodine-azide reaction. In some experiments with normal rabbit whole blood and serum it was found that the latter had a greater catalytic activity than the former. It has not yet been possible to determine whether we are dealing with species differences or with effects of ageing of the blood fluids.

### SUMMARY.

Sera from rats bearing various transplant tumours have generally much reduced capacities for catalysing the decomposition of iodine-azide reagent as compared with normal sera. There is a highly significant negative linear correlation relating log tumour per cent and log 100'*N*. Similar correlations hold for oxalated whole blood and fresh serum from tumour-bearing rats.

A polarographic proteose test employed as a " control " for the iodine-azide reaction proved to be statistically unreliable. Only very small correlations (without significance) were found between tumour sera proteose wave heights and tumour percentage or serum 100′$N$ values. However the test did show a significant linear negative correlation between serum proteose and 100′$N$ values for normal animals.

Thanks are due to Professor H. N. Green for his interest in this work and to the British Empire Cancer Campaign for a grant.

REFERENCES.

Bradford Hill, A.—(1952) 'Principles of Medical Statistics,' 5th ed. London (The Lancet, Ltd.).
Feigl, F.—(1939) 'Qualitative Analysis by Spot Tests,' 2nd ed. New York (Nordeman Publishing Co. Inc.).
Green, H. N., and Jenkinson, C. N.—(1934) *Brit. J. exp. Path.*, **15**, 1.
Kolthoff, I. M., and Lingane, J. J.—(1953) 'Polarography', **2**, pp. 849–876, 2nd ed. New York and London. (Interscience Publishers).
Løvtrup, S.—(1951) *C.R. des trav. du Lab. Carlsberg, Sér chim.*, **27**, 63.
Moroney, M. J.—(1953) 'Facts from Figures,' pp. 271–319, 2nd ed. London (Penguin Books, Ltd.).
Munro, L. A., and Boyd, D. J.—(1951) *Canad. J. Chem.*, **29**, 1115.
Raschig, F.—(1915) *Ber. dtsch. chem. Ges.*, **48**, 2088.
Robinson, A. M.—(1948) *Brit. J. Cancer*, **2**, 360.
Rosenthal, R. L.—(1949) *Science*, **110**, 43.
Umbreit, W. W., Burris, R. H., and Stauffer, J. F.—(1951) 'Manometric Techniques and Tissue Metabolism,' 2nd ed. Minneapolis (Burgess Publishing Co.).
Whitman, D. W., and Whitney, R. M.—(1953) *Analyt. Chem.*, **25**, 1523.
Winzler, R. J.—(1953) *Plasma Proteins in Cancer in Advances in Cancer Research*, **1**, 503.